# Neutrophil Elastase Alters the Murine Gut Microbiota Resulting in Enhanced *Salmonella* Colonization

**DOI:** 10.1371/journal.pone.0049646

**Published:** 2012-11-14

**Authors:** Navkiran Gill, Rosana B. R. Ferreira, L. Caetano M. Antunes, Benjamin P. Willing, Inna Sekirov, Fatimah Al-Zahrani, Martin Hartmann, B. Brett Finlay

**Affiliations:** 1 Michael Smith Laboratories, The University of British Columbia, Vancouver, British Columbia, Canada; 2 Department of Microbiology and Immunology, The University of British Columbia, Vancouver, British Columbia, Canada; 3 Molecular Ecology, Agroscope Reckenholz-Tänikon Research Station ART, Zurich, Switzerland; 4 Soil Sciences, Swiss Federal Research Institute WSL, Birmensdorf, Switzerland; Institut National de la Recherche Agronomique, France

## Abstract

The intestinal microbiota has been found to play a central role in the colonization of *Salmonella enterica* serovar Typhimurium in the gastrointestinal tract. In this study, we present a novel process through which *Salmonella* benefit from inflammatory induced changes in the microbiota in order to facilitate disease. We show that *Salmonella* infection in mice causes recruitment of neutrophils to the gut lumen, resulting in significant changes in the composition of the intestinal microbiota. This occurs through the production of the enzyme elastase by neutrophils. Administration of recombinant neutrophil elastase to infected animals under conditions that do not elicit neutrophil recruitment caused shifts in microbiota composition that favored *Salmonella* colonization, while inhibition of neutrophil elastase reduced colonization. This study reveals a new relationship between the microbiota and the host during infection.

## Introduction

The gastrointestinal tract is populated by a complex community of microbes with important biological functions. This community, referred to as the microbiota, is a first line of defense against invading pathogens [1], [2], [3]. During infection, gastrointestinal pathogens need to circumvent the defense mechanisms deployed by the host and also the significant competitive barrier presented by the microbiota. Disruption of the intestinal microbiota through the use of antibiotics has been known for several decades to increase susceptibility to enteric infection, emphasizing the importance of intestinal commensals for protection against pathogens. The protection conferred by the microbiota has been termed “colonization resistance”, a term used to generally describe all mechanisms by which the microbiota protects its host from pathogens [4], [5].

*Salmonella enterica* serovar Typhimurium is an enteric pathogen that can successfully circumvent the protective functions of the intestinal microbiota in humans to cause gastroenteritis [6], [7]. The mechanisms involved are poorly understood, although recent studies have shed some light onto this phenomenon. Stecher *et al*. have shown that inflammation causes shifts in the intestinal microbiota that reduce the growth of commensals and favor *Salmonella* growth in a murine infection model [8]. *Salmonella* mutants that are unable to cause inflammation do not affect gut microbiota composition and are outcompeted by the microbiota. More recently, a mechanism through which intestinal inflammation favors *Salmonella* growth was proposed; Winter *et al.* showed that the production of reactive oxygen species as a consequence of gut inflammation generated an alternative electron acceptor that could be preferentially used by *Salmonella*, allowing it to outcompete the gut microbiota [9]. These recent findings strengthen the concept that *Salmonella* benefits from intestinal inflammation as part of its pathogenesis.

One of the first lines of the innate immune response is the recruitment of neutrophils [10]. These are phagocytic cells whose main function is to eliminate pathogens. As such, their recruitment, as well as inflammation in general, has been classically seen as detrimental to pathogen growth. We have recently shown that neutrophils are recruited to the intestinal lumen during *Salmonella* infection using a gastroenteritis model that involves treatment of mice with a low dose of streptomycin prior to infection [11]. This phenomenon is dependent upon the *Salmonella* Pathogenicity Island 1 (SPI-1), and is accompanied by a disruption of the intestinal microbiota, with a reduction of the total numbers of bacteria present in the gut as well as significant changes in the microbial composition of this community. This suggests that neutrophil recruitment has a role in shaping the composition of the microbiota during *Salmonella* infection. Here, we provide direct evidence that this is indeed the case; Neutrophil recruitment can be directly linked to alterations of the intestinal microbiota during infection. Additionally, we show that the mechanism for this relies on the production of a single enzyme by neutrophils, namely elastase, and that elastase-elicited microbiota disruption facilitates *Salmonella* gut colonization. This study unveils a novel mechanism through which *Salmonella* benefits from an innate immune host function to gain an advantage against the competing intestinal microbiota and facilitate colonization.

## Results

### *Salmonella ΔaroAΔinvA* and *ΔaroAΔssaR* Mutants have a Significant Defect in Cecum Colonization and Neutrophil Influx

**Figure 1:**
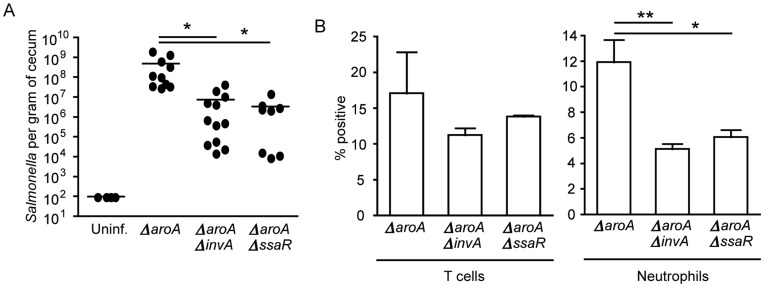
Infection with *ΔaroA Salmonella* results in extensive gut colonization and neutrophil recruitment. Six- to eight-week-old female mice were treated with 450 mg/L of streptomycin for 2 days in their drinking water. After antibiotic withdrawal, mice were infected with 2.7×10^8^ colony-forming units (CFUs) of the indicated *Salmonella* strain. A) *Salmonella* colonization was enumerated at five days post infection by plating serial dilutions of cecum homogenates on LB plates supplemented with 100 µg/mL of streptomycin. The limit of detection for this assay was 100 CFUs. B) Ceca were harvested at five days post infection and single cell suspensions were obtained. Staining and flow cytometry analyses were used to determine percentages of T cells (CD45+CD3+NK1.1−) and neutrophils (CD45+GR1+MPO+). Four mice per group were used, and experiments were repeated at least three times. * indicates *p*<0.05, **p<0.01.

To gain insights into the relationship between neutrophil recruitment and microbiota composition in the mammalian gut, as well as to investigate the mechanism through which neutrophils impact on intestinal commensals, we performed a more detailed analysis of the effect of neutrophil influx on the intestinal microbiota. We used the low-dose streptomycin model previously described, where mice were treated with streptomycin in drinking water (450 mg/L) over two days and then infected with *Salmonella* [12]. Because infection with wild-type *Salmonella* causes overt inflammation and tissue damage, we used a *Salmonella aroA* mutant, which is attenuated in a murine infection model [13], [14]. This allowed us to observe more subtle effects of infection and neutrophil influx on the intestinal microbiota that would have been difficult to study during the aggressive infection caused by wild-type *Salmonella*. Following streptomycin treatment, mice were infected with *ΔaroA Salmonella* and, after 5 days of infection, mice were sacrificed and bacterial loads in the cecum as well as neutrophil influx were assessed. As expected, all mice infected with the *ΔaroA* strain were colonized to high levels (Figure 1A) and there was a marked influx of neutrophils into the intestinal tissue, with these cells accounting for over 10% of all cells present in the samples (Figure 1B), whereas in uninfected animals neutrophils were undetectable (data not shown). Our previous work showed that mutants in *Salmonella* pathogenicity islands (SPI) 1 and 2 cause reduced neutrophil influx after streptomycin treatment and *Salmonella* infection [11]. In order to determine if this phenotype also occurred in the *ΔaroA* strain, we infected two groups of streptomycin-treated mice with either a *ΔaroAΔinvA* or a *ΔaroAΔssaR* strain and assessed bacterial loads and neutrophil influx. We found that both SPI-1 (*ΔinvA*) and SPI-2 (*ΔssaR*) mutations on an *ΔaroA* background caused significantly lower bacterial colonization (Figure 1A) as well as significantly lower numbers of neutrophils in the cecum of infected mice (Figure 1B), similar to what has been previously reported with infections with the wild-type strain.

### The *ΔaroA* but not *ΔaroAΔinvA* or *ΔaroAΔssaR Salmonella* Strains Disrupt Gut Microbiota Composition

**Figure 2:**
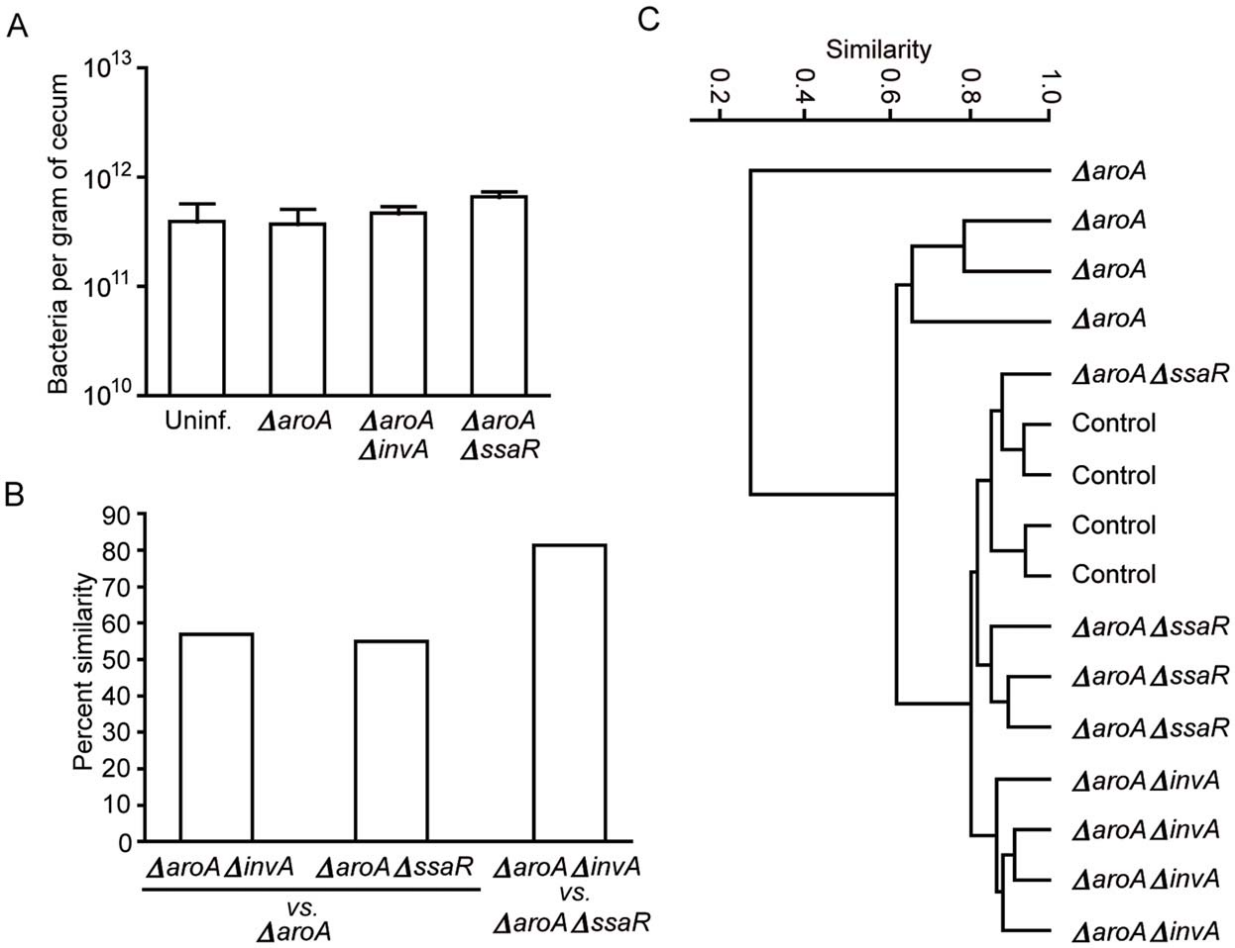
The gut microbiota composition of *ΔaroA*-infected mice is significantly altered compared to *ΔaroAΔinvA-* and *ΔaroAΔssaR*-infected mice. Six- to eight-week-old female mice were treated with 450 mg/L of streptomycin for 2 days in their drinking water. After antibiotic withdrawal, mice were infected with 2.7×10^8^ CFUs of the indicated strain. A) SYBR staining was utilized to determine the total number of bacteria in cecum samples at day 5 post infection; No significant differences were found. B) and C) Bacterial 16s rRNA genes was amplified from fecal samples at day 5 post infection for TRFLP analysis. B) graph representing percent similarity of microbiota between groups C) dendrogram showing separation of treatment groups based on changes in microbial composition. *ΔaroA*-infected mice were significantly different in their microbial composition compared to *ΔaroAΔinvA-* and *ΔaroAΔssaR-*infected and control uninfected mice. Four mice per group were used, and experiments were repeated at least three times. Data from one representative experiment is shown.

We next sought to determine the impact of infection with the SPI-1 and SPI-2 mutant strains on the composition of the gut microbiota. First, we determined total bacterial counts in feces from mice treated with low-dose streptomycin and infected with each of the three strains. We found that none of the strains (*ΔaroA*, *ΔaroAΔinvA* or *ΔaroAΔssaR*) caused a significant change in the total number of bacteria colonizing the gastrointestinal tract (Figure 2A). This represents another advantage of using the *ΔaroA* background, since phenotypes related to the intestinal microbiota can be attributed to a shift in composition as opposed to a decrease in general microbial numbers in this model. When we analyzed the composition of the gut microbiota of mice infected with each of the three strains, we found that only infection with the *ΔaroA* strain caused a significant change. Infection with *ΔaroAΔinvA* or *ΔaroAΔssaR Salmonella* resulted in a microbiota that was similar to that of uninfected mice using Terminal Restriction Fragment Length Polymorphism (TRFLP) analysis and Bray-Curtis metrics (Figures 2B, 2C).

### Neutrophil Depletion Eliminates the Shift in the Intestinal Microbiota Composition Caused by Infection with *ΔaroA Salmonella*

**Figure 3:**
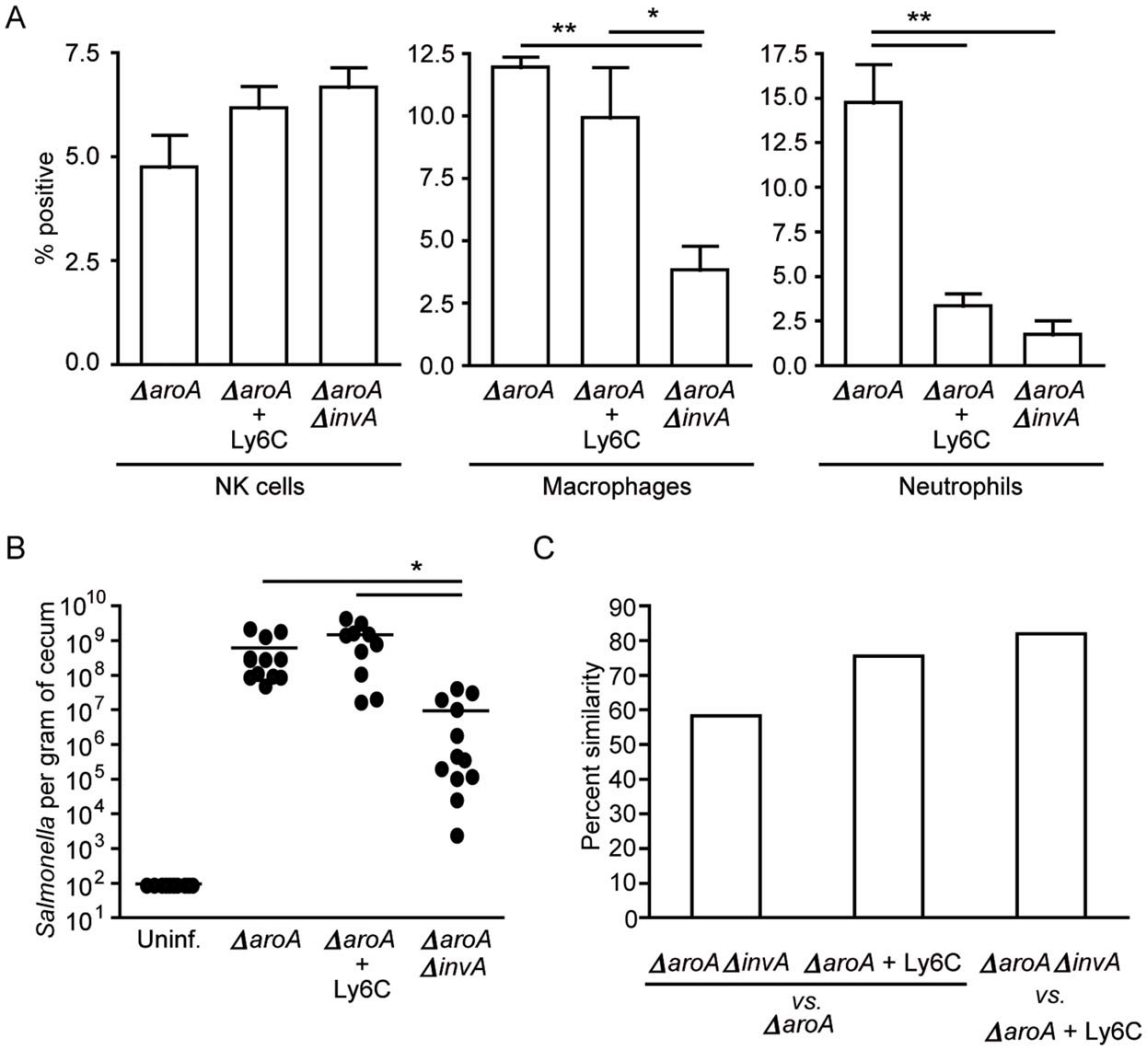
Neutrophil depletion in *ΔaroA*-infected mice eliminates the shift in microbiota composition caused by *Salmonella* infection. Six- to eight-week-old female mice were treated with 450 mg/L of streptomycin for 2 days in their drinking water. After antibiotic withdrawal, mice were infected with 2.7×10^8^ cells of the indicated strain. Neutrophils were depleted by injecting Ly6c antibody intraperitoneally one day before infection and 1 and 3 days post infection. A) Ceca were harvested at five days post infection and single cell suspensions were obtained. Staining and flow cytometry analyses were used to confirm that neutrophils were significantly decreased in Ly6c-treated mice. NK cells (CD45+NK1.1+CD3-), macrophages (CD45+CD11c+CD11b+) and neutrophils (CD45+GR1+MPO+) were analyzed. B) *Salmonella* colonization levels were determined five days post infection by plating serial dilutions of cecum homogenates on LB plates supplemented with 100 µg/ml of streptomycin. C) Bacterial 16s rRNA genes were amplified from fecal samples at day 5 post infection for TRFLP analysis, graph shows percent similarity of microbiota between treatment groups. Gut microbiota composition of *ΔaroA*-infected mice was significantly different compared to *ΔaroAΔinvA-*infected mice or neutrophil depleted, *ΔaroA*-infected mice. Four mice per group were used, and experiments were repeated at least three times. * indicates *p*<0.05, **p<0.01.

The fact that mutations in SPI-1 or SPI-2 genes dampened neutrophil recruitment during infection could be simply a consequence of reduced pathogen load in the gastrointestinal tract. Additionally, although the absence of neutrophil recruitment during infection with the SPI mutants correlated with the lack of microbiota disruption, it was still possible that the effect of wild-type infection on microbiota composition was uncoupled from the extent of neutrophil infiltration. However, we have previously shown that at day 5 post infection with a ***Δ*** *invA* strain there is significant inflammation, yet no changes in the microbiota profile were observed [11]. This suggests that the absence of microbiota disruption during infections with SPI-1 or SPI-2 mutants in both our previous and current studies is not solely a consequence of reduced inflammatory influx but, rather, specific to neutrophil infiltration. To test this hypothesis we infected mice with *ΔaroA Salmonella* and treated them with an anti-mouse Ly6G/Ly6C monoclonal antibody to deplete neutrophil populations [15]. Indeed, this treatment resulted in a significant decrease in the number of neutrophils in the cecum while not affecting numbers of other cell populations (natural killer cells, macrophages; Figure 3A). Interestingly, depletion of neutrophils from the intestinal tract significantly affected the composition of the intestinal microbiota (Figure 3C), despite having no significant effect on pathogen loads (Figure 3B). The resulting microbiota composition was similar to that achieved during infection with the *ΔaroAΔinvA* strain (Figure 3C). This experiment establishes for the first time a direct link between neutrophil recruitment and microbiota disruption.

### Inhibition of a Single Neutrophil Function Recapitulates the Effect of Neutrophil Depletion on Gut Microbiota Composition

**Figure 4:**
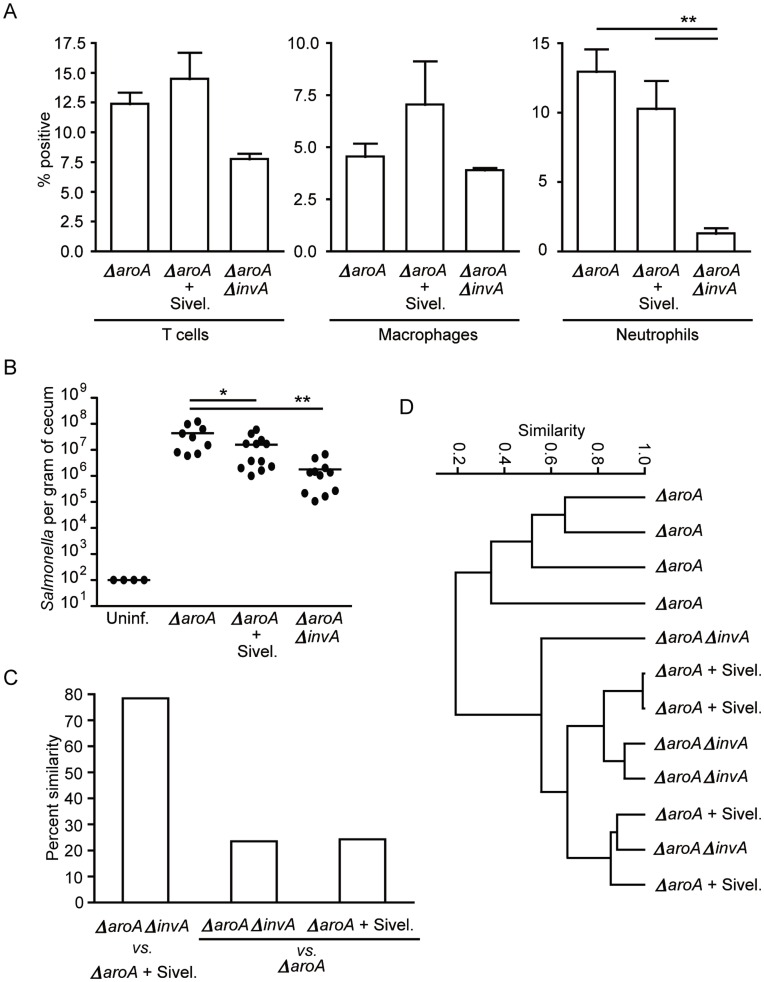
Neutrophil elastase inhibition during *ΔaroA Salmonella* infection eliminates the effect of infection on gut microbiota composition. Six- to eight-week-old female mice were treated with 450 mg/L of streptomycin for 2 days in their drinking water. After antibiotic withdrawal, mice were infected with 2.7×10^8^ CFUs of the indicated strain. Neutrophil elastase was inhibited by injection of Sivelestat sodium hydrate (Sivel.) intraperitoneally twice a day on days -1, 0, 1, 2, 3, and 4 post infection. A) Ceca were harvested at five days post infection and single cell suspensions were obtained. Staining and flow cytometry analyses were used to confirm that neutrophils were not decreased in mice treated with the neutrophil elastase inhibitor. T cells (CD45+CD3+NK1.1-), neutrophils (CD45+GR1+MPO+) and macrophages (CD45+CD11c+CD11b+) were analyzed. B) *Salmonella* colonization levels were determined five days post infection by plating serial dilutions of cecum homogenates on LB plates supplemented with 100 µg/ml of streptomycin. Outliers were detected using the Grubbs’ test and removed from the dataset. C) and D) Bacterial 16s rRNA genes were amplified from fecal samples at day 5 post infection for TRFLP analysis. C) graph showing percent similarity of microbiota between treatment groups D) dendrogram showing the separation of treatment groups based on changes in microbial composition. *ΔaroA-*infected mice receiving neutrophil elastase inhibitor showed a significantly different gut microbial composition compared to untreated mice but similar to *ΔaroAΔinvA*-infected mice. Four mice per group were used, and experiments were repeated at least three times. Outliers were removed from data sets using Grubbs’ test. * indicates *p*<0.05, **p<0.01.

After determining that neutrophil recruitment during infection was in fact the determining factor in whether or not the gut microbiota was affected, we sought to identify the mechanism through which neutrophil activity impacted on intestinal commensals. During inflammation, neutrophils are known to produce a serine protease of broad substrate specificity (elastase) that has antimicrobial activity [16]. Therefore, we hypothesized that neutrophil elastase would be a good candidate for the disruption of microbial composition in the gastrointestinal tract elicited by neutrophil influx. To test this hypothesis, we treated *ΔaroA*-infected mice with a neutrophil elastase inhibitor (Sivelestat sodium salt hydrate – Sivel) and evaluated the effect of treatment on pathogen colonization as well as gut microbiota composition. In agreement with our hypothesis, treatment with the neutrophil elastase inhibitor during infection with *ΔaroA Salmonella* resulted in a microbiota profile that was similar to the microbiota of *ΔaroAΔinvA* infected mice (also lack luminal neutrophil elastase due to a lack of neutrophil recruitment) (Figures 4C, 4D). Furthermore, this treatment resulted in a significant reduction in *Salmonella* colonization compared to *ΔaroA* infected mice not treated with neutrophil elastase inhibitor, (Figure 4B), suggesting an important role for microbiota composition in *Salmonella* colonization success. Treatment with the elastase inhibitor did not significantly alter neutrophil numbers in the gut (Figure 4A), supporting the concept that the effect is specific to the neutrophil elastase activity.

### Recombinant Neutrophil Elastase Treatment can Recapitulate Microbiota Disruption and *Salmonella* Colonization during Infection with *ΔaroAΔinvA Salmonella*

**Figure 5:**
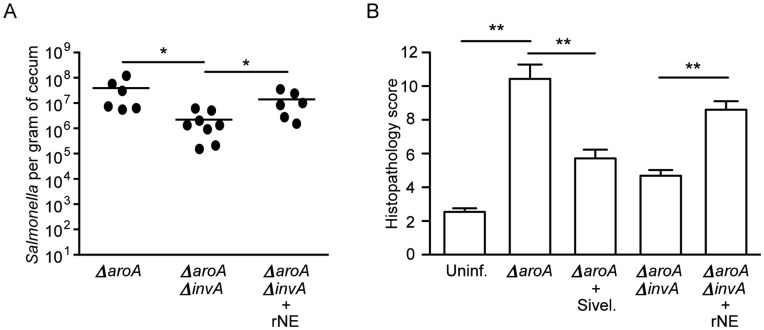
Recombinant neutrophil elastase delivery to mice infected with *ΔaroAΔinvA Salmonella* results in increased colonization and significant changes in histopathology. Six- to eight-week-old female mice were treated with 450 mg/L of streptomycin for 2 days in their drinking water. After antibiotic withdrawal, mice were infected with 2.7×10^8^ CFUs of the indicated strain. Recombinant neutrophil elastase was injected orally and intraperitoneally once a day on days 0, 1, 2, 3, and 4 post infection. A) *Salmonella* colonization was determined at five days post infection by plating serial dilutions of cecum homogenates on LB plates supplemented with 100 µg/ml of streptomycin. B) Pathology scores of cecal tissue 5 days post infection. * indicates *p*<0.05, ** indicates p<0.01.

**Figure 6:**
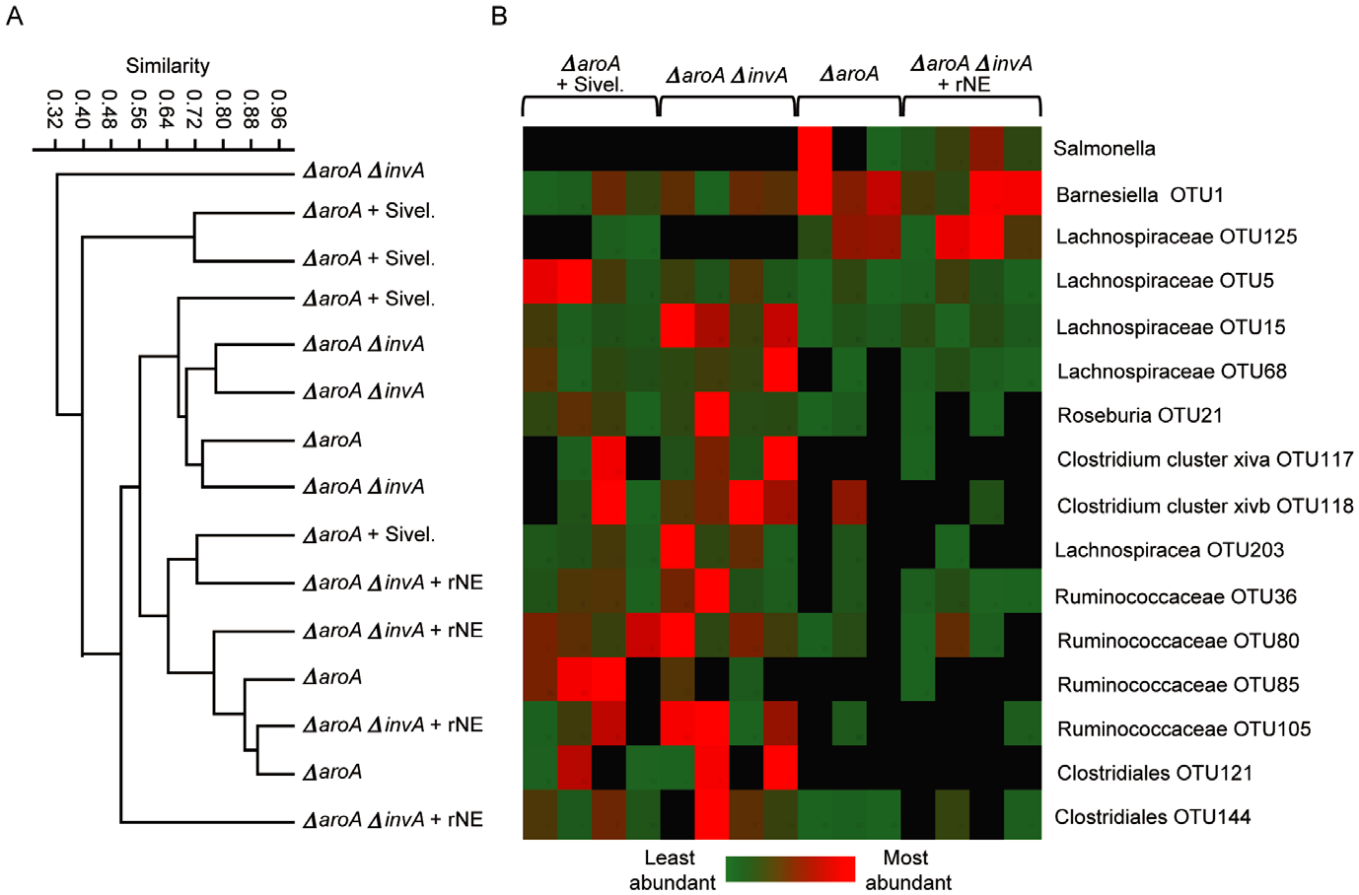
16S rRNA gene pyrosequencing revealed several OTUs whose abundance correlates with neutrophil elastase activity in the gut during infection. Six- to eight-week-old female mice were treated with 450 mg/L of streptomycin for 2 days in their drinking water. After antibiotic withdrawal, mice were infected with 2.7×10^8^ cells of the indicated strain. Bacterial 16S rRNA genes were amplified with bar-coded primers from DNA isolated from fecal samples at day 5 post infection. A) Microbial profiles were compared by cluster analysis at the OTU level using Bray-Curtis metrics. B) Heat map showing relative abundance of OTUs that differed between groups.

In order to obtain further evidence that neutrophil elastase is responsible for the microbiota disruption observed during *ΔaroA Salmonella* infection, we infected mice with the *ΔaroAΔinvA* strain and studied the effect of administration of recombinant neutrophil elastase on microbiota composition and pathogen colonization. Successful delivery of neutrophil elastase was confirmed through western blots of cecal samples (data not shown). We examined colonization by *Salmonella* in neutrophil elastase treated mice and found it to be significantly increased (Figure 5A). Furthermore, when we examined pathology of ceca in the various treatment groups we found that treatment of *ΔaroAΔinvA* infected mice with recombinant neutrophil elastase resulted in increased pathology, while inhibition of neutrophil elastase in *ΔaroA* infected mice significantly reduced overt pathology (Figure 5B). To identify bacterial taxa affected by infection and neutrophil infiltration we assessed changes in the gut microbiota using 16S rRNA gene pyrosequencing. A total of 183,656 quality sequences from 82 samples were analyzed with an average of 2243+/−864 (+/− SD). It was noted that mice that had neutrophil elastase in their ceca showed a drastic change in select bacterial groups. Specifically, OTUs belonging to the *Ruminococcaceae* and *Lachnospiraceae* families were significantly reduced in neutrophil elastase treated mice (Figure 6B). Furthermore, *ΔaroAΔinvA* infected and *ΔaroA* + sivelestat sodium salt hydrate treated (neutrophil elastase deficient) mice possessed similar microbiota compared to groups that had neutrophil elastase (Figure 6A). These results confirm neutrophil elastase as the host factor responsible for gut microbiota disruption during infection and suggest that neutrophil influx and elastase production is responsible for the control of pathogen loads in host intestinal tissue during *Salmonella* infection.

### The Effects of Inhibition of Neutrophil Elastase Activity or Treatment with Recombinant Neutrophil Elastase on *Salmonella* Colonization can be Recapitulated during Infection with Wild-type *Salmonella*

**Figure 7:**
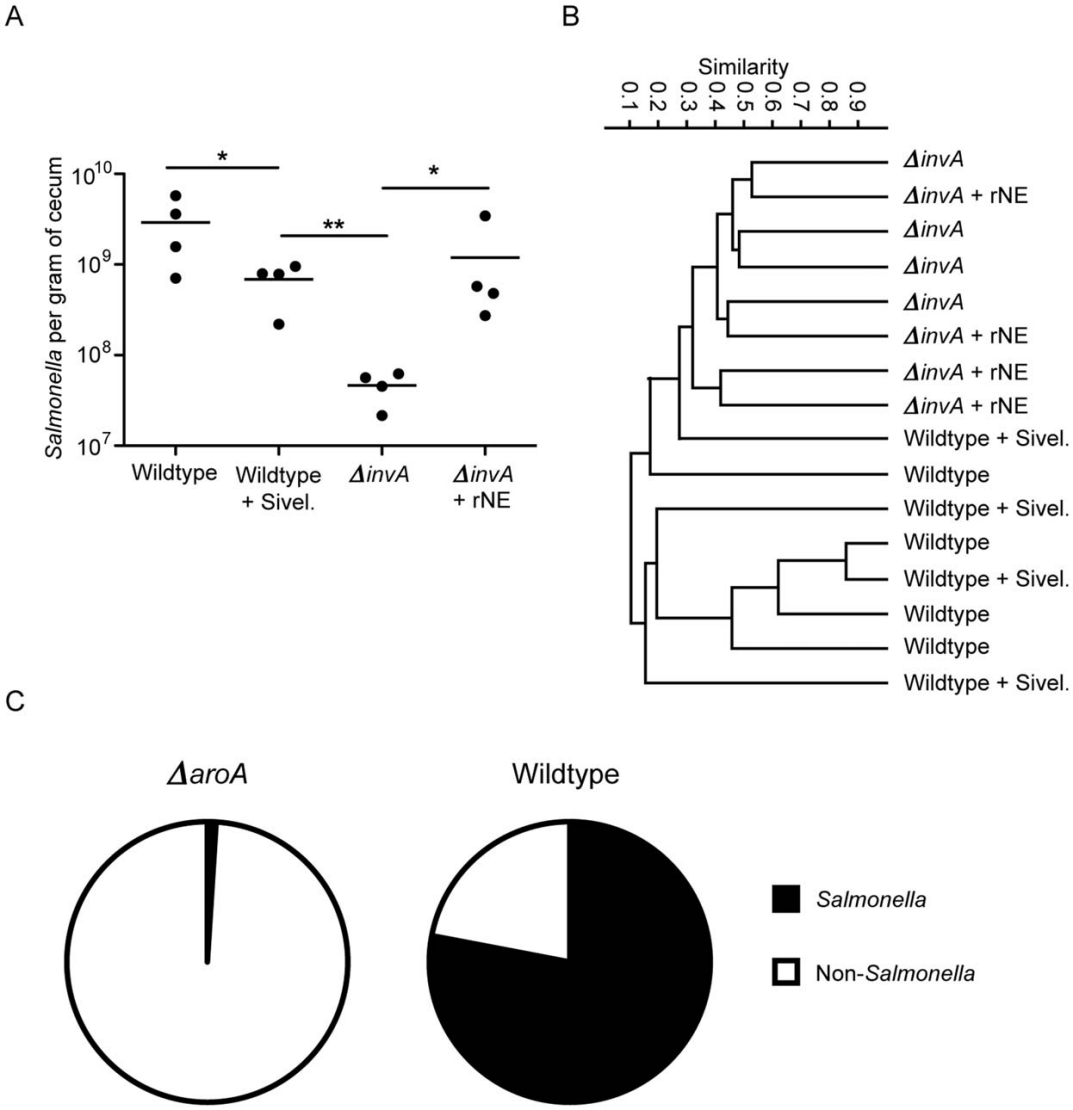
Modulation of neutrophil elastase activity during wild-type infection alters pathogen loads but do not significantly affect microbiota composition. Six- to eight-week-old female mice were treated with 450 mg/L of streptomycin for 2 days in their drinking water. After antibiotic withdrawal, mice were infected with 2.7×10^8^ cells of the indicated wild type strain. A) *Salmonella* colonization levels were determined three days post infection by plating serial dilutions of cecum homogenates on LB plates supplemented with 100 µg/ml of streptomycin. B) Bacterial 16S rRNA gene fragments were isolated from fecal samples at day 5 post infection and amplified using 33 nucleotide-bar-coded primer pairs. 454 pyrosequencing reads were analyzed by cluster analysis using Bray-Curtis metrics of microbial profiles. C) *Salmonella* abundance was assessed by 454 pyrosequencing of bacterial 16S rRNA gene fragments isolated from feces of mice infected with either wild-type or *ΔaroA Salmonella*.

Because infection with wild-type *Salmonella* causes overt inflammation and tissue damage, all experiments aforementioned were performed using a mildly attenuated *Salmonella* strain (*aroA* mutant) [13], [14]. Although this allowed us to observe subtle effects of infection and neutrophil influx on the intestinal microbiota, it still remained to be determined whether the findings reported herein were relevant during infection with wild-type *Salmonella* (SL1344). Therefore, we first infected mice with wild-type *Salmonella* and treated the animals with neutrophil elastase inhibitor or recombinant neutrophil elastase as described before. Supporting the notion that neutrophil elastase is an important factor allowing *Salmonella* to fully colonize its host, treatment with the neutrophil elastase inhibitor significantly decreased *Salmonella* loads in the cecum (Figure 7A). Conversely, we infected mice with a *ΔinvA* strain and treated the animals with recombinant neutrophil elastase. As we expected, treatment with elastase caused an expressive (and statistically significant) increase in *Salmonella* loads in the cecum (Figure 7A). In addition to studying the effect of treatments on *Salmonella* colonization, we also analyzed the intestinal microbial composition in each of the treatments. Treatment with neutrophil elastase inhibitor or recombinant neutrophil elastase did not significantly affect intestinal microbiota composition (Figure 7B). However, it is important to note that infection with wild-type *Salmonella* is overwhelming, and microbiota characterization through 16S rRNA gene sequencing shows that most hits are from *Salmonella* itself (Figure 7C). Therefore, it is impossible to profile the intestinal microbial community and the effects of neutrophil elastase treatments during wild-type infection reliably. Nevertheless, even in the absence of detectable microbiota changes during *Salmonella* infection, our cecum colonization data supports the role of neutrophil elastase as an important factor during *Salmonella* infection. We next attempted to re-capitulate our model in neutrophil elastase knockout mice. However, the microbiota of the knockout mice is drastically different from the microbiota of wild-type mice, and is not similar to the microbiota of mice that have neutrophil elastase inhibited (data not shown). Although we did observe that the neutrophil elastase deficient mice were slightly less susceptible to *Salmonella* colonization, these were not statistically significant changes (data not shown). Hence, it is impossible for us to use these animals to ask questions relevant to our research.

### Neutrophil Elastase can Alter Host Gut Microbiota Independently of Enteric Infection

**Figure 8:**
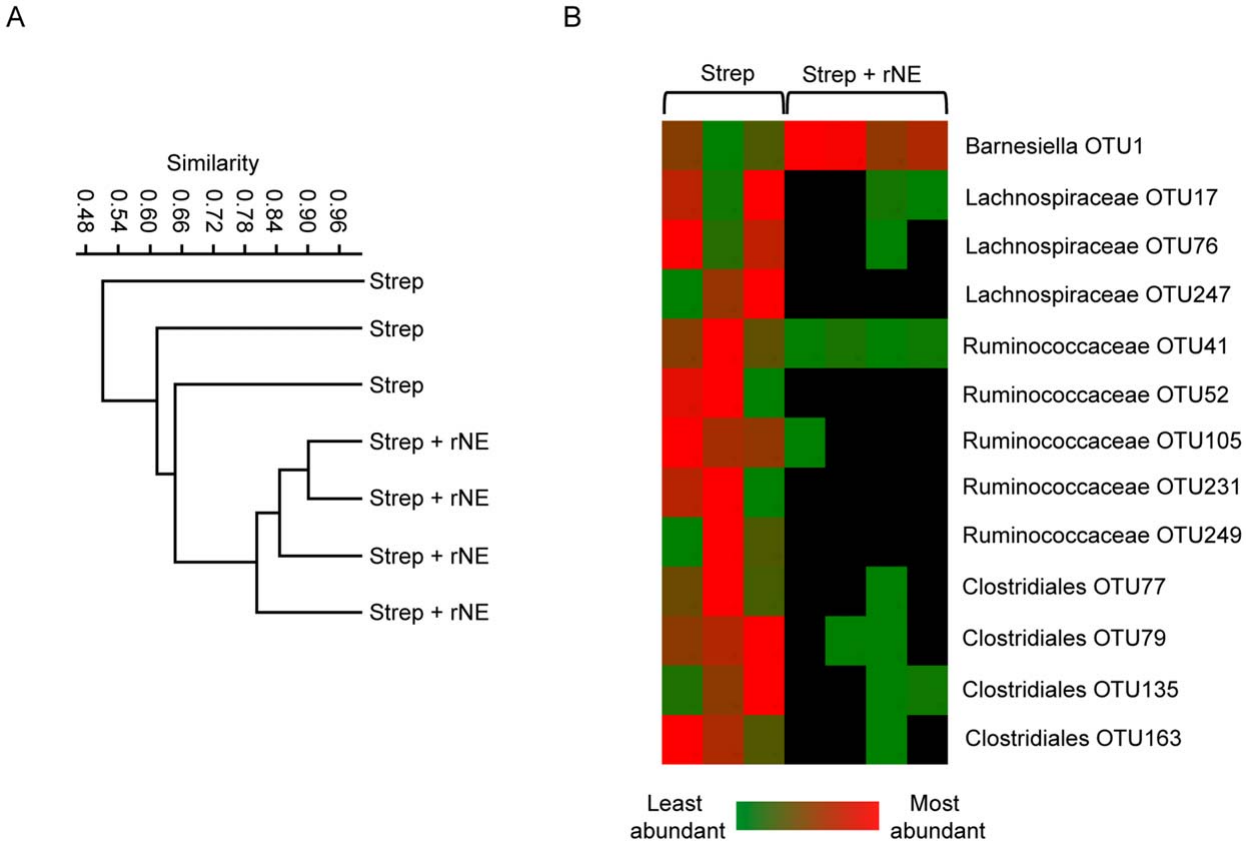
Recombinant neutrophil elastase treatment causes changes in the gut microbiota composition in the absence of infection. Six- to eight-week-old female mice were treated with 450 mg/L of streptomycin for 2 days in their drinking water. After antibiotic withdrawal recombinant neutrophil elastase was given both orally and intraperitoneally every day for 4 days. Fecal samples were collected at day 4 post treatment. A) 454 pyrosequencing reads were analyzed by cluster analysis using Bray-Curtis metrics of microbial profiles. B) Heat map showing relative abundance of different OTU groups.

Our results show that neutrophil elastase can impact on the gut microbiota during *Salmonella* infection. Next we wanted to address whether elastase itself was enough to cause shifts in the intestinal microbiota in the absence of an additional disturbance, such as enteric infection. To do so, we treated uninfected animals with recombinant elastase and analyzed the effect of treatment on gut microbiota composition through 16s rRNA gene sequencing. Figure 8 shows that recombinant neutrophil elastase significantly altered gut microbiota composition. A dendogram using Bray-Curtis metrics depicts that mice given recombinant neutrophil elastase had significantly different microbial profiles compared to untreated mice (Figure 8A). Furthermore, a heat map of the relative abundance of OTUs that were significantly affected by elastase (Figure 8B) shows that members of the same bacterial families (*Ruminococcaceae* and *Lachnospiraceae*) were eliminated or depleted in treated mice as those observed in the infection model. Altogether, our data support the notion that neutrophil influx, and more specifically the neutrophil elastase activity that accompanies it, can have a significant impact on gut microbiota composition and therefore, susceptibility to enteric disease.

## Discussion

In this work, we studied the role of neutrophils during *Salmonella* infection with the purpose of understanding whether the host immune response initiated during infection could cause disruption of the intestinal microbiota composition and impact *Salmonella* colonization. Indeed, we found that neutrophil recruitment is directly associated with changes to the gut microbiota profile. Specifically, we showed that disruption of the gut microbiota by neutrophils is a consequence of a single protein, namely neutrophil elastase. Neutrophil elastase in turn, assists *Salmonella* in successful colonization of the gut. The intestinal microbiota represents a powerful barrier against invading pathogenic microorganisms [3], [5]. In healthy individuals, this barrier can keep most pathogens out; however, during disturbances such as an underlying illness or antibiotic treatment this barrier is compromised. Much the same way that the intestinal microbiota has evolved ways to protect its host from external attack, pathogens have also devised sophisticated mechanisms to circumvent this obstacle. *Salmonella enterica* serovar Typhimurium is one such pathogen, which can colonize the human gastrointestinal tract to cause severe, although self-limiting, gastroenteritis [6], [7]. Although *Salmonella* can successfully overcome the host’s gut microbiota and colonize the intestinal tract, it has been known since the 1950’s that streptomycin treatment increases susceptibility to infection [4], [17]. It has generally been assumed that this is due to killing of the intestinal microbiota and therefore, decreasing competition for nutrients and binding sites. Although this is probably true, we now know that more sophisticated mechanisms of pathogen control are in place. For instance, the intestinal microbiota is involved in inducing the production of many antibacterial molecules by the host that assist in protecting it from invasion [18], [19].

Despite all the mechanisms of defense present in the intestinal tract, obviously under some conditions infection still occurs. When this is the case, many other aspects of the innate immune system come into play as secondary lines of defense against the incoming pathogen. Pathogenic microorganisms are quickly sensed through cell-surface receptors on the intestinal epithelium as well as sampling by immune cells residing in the lamina propria [5], [10]. This initiates a cascade of signaling events that culminate with the influx of many inflammatory cells whose function is to control the infection before it can spread. Although under most conditions inflammation does benefit the host, some pathogenic microbes have adapted to take advantage of the inflammatory process. For instance, *Salmonella enterica* and *Citrobacter rodentium* have been shown to use inflammation to outcompete the intestinal microbiota [8], [20]. Multiple studies have shown that several mechanisms are involved in this process. Winter *et al*. have recently shown that gut inflammation generates reactive oxygen species that, in turn, react with sulphur compounds present in the gut lumen to generate tetrathionate, which can be used by *Salmonella* as a terminal electron acceptor during anaerobic respiration [9]. Due to the anoxic nature of the mammalian gut, most gut commensals use fermentation as a means to obtain energy and consequently will not respire tetrathionate. Therefore, the presence of this electron acceptor provides *Salmonella* with a significant selective advantage over the intestinal microbiota. There are also other inflammation-elicited events that are involved in the ability of *Salmonella* to outgrow the microbiota. For instance, Stelter *et al.* have shown that enteric infection and the subsequent inflammation induced the expression of the C-type lectin RegIIIβ [21]. RegIIIβ has antimicrobial activity and can kill intestinal commensals without affecting *Salmonella* due to structural differences in its lipopolysaccharide. Therefore, RegIIIβ production during infection represents another mechanism through which *Salmonella* can benefit from inflammation.

We have recently shown that *Salmonella* infection in a low-dose, streptomycin pre-treatment model (a gastroenteritis model) results in a significant influx of inflammatory cells, many of which are neutrophils [11]. Due to the interactions between *Salmonella* and the inflammatory process noted above, we examined the role of neutrophils during infection. While doing so, we found that neutrophil recruitment is positively correlated with significant changes in the composition of the intestinal microbiota. Here we show that neutrophil recruitment is directly responsible for the shifts in gut microbiota composition. Depletion of neutrophils during *Salmonella* infection abolished the effect on the intestinal microbiota. More specifically, we linked this activity to the production of neutrophil elastase by showing that elastase inhibition eliminated the phenotype observed and administration of recombinant enzyme caused changes in the microbiota during Δ*aroA*Δ*invA* infection that were similar to the effects of wild-type (Δ*aroA*) infection. Since shifts in the intestinal microbiota have been associated with increased susceptibility to *Salmonella* infection [12], [22], we also studied the effects of neutrophil elastase on *Salmonella* host colonization. Depletion of neutrophils did not affect infection with the Δ*aroA Salmonella* strain, while inhibition of elastase did cause significant decreases in *Salmonella* colonization. Furthermore, administration of recombinant elastase to Δ*aroA*Δ*invA* caused a significant increase in bacterial burden in the intestinal tract. The fact that we did not observe a decrease in colonization upon neutrophil depletion is not all that surprising; neutrophil recruitment is an important aspect of host defense against invading pathogens. They are efficient phagocytic cells and also release a plethora of other immune factors that may play an important role in controlling *Salmonella* colonization. Although elimination of neutrophils did show significant changes to the microbiota profile, this alone was not sufficient to inhibit colonization. On the other hand, we did not observe an increase in colonization in the absence of neutrophils, which is attributed to the already saturated numbers of *Salmonella* in the gut. Although neutrophil recruitment is an important host response to inhibit pathogens, it can also benefit S*almonella* under certain conditions. We carried out this infection model using a wild-type strain of Salmonella and found that as in the Δ*aroA* infection model changes in colonization could be attributed to the presence or absence of neutrophil elastase in the gut during infection. However, during infection with wild-type *Salmonella* the burden is so high that changes in the microbiota could not be accurately monitored. Hence, our data showing that administration of recombinant neutrophil elastase to streptomycin pre-treated and infected mice favors *Salmonella* growth and that inhibition of neutrophil elastase reduces colonization supports the notion that neutrophil elastase activity in the gut is beneficial to *Salmonella*.

The exact mechanisms through which neutrophil elastase favors *Salmonella* growth are unknown at this time. In an effort to shed light on this, we have characterized the impact of neutrophil elastase on gut microbiota composition and have found several bacterial families that are changed in mice with neutrophil elastase activity. Specifically, we have found that neutrophil elastase activity is associated with an increase in *Barnsiella* numbers and a decrease or loss of several *Lachnospiraceae* and *Ruminococcaceae* families in the gastrointestinal tract. The loss of these members of the microbiota in the presence of neutrophil elastase suggests that they may play a role in actively competing with *Salmonella* for nutrients or specific colonization niches. Hence, when neutrophil elastase is inhibited and these groups are present in the gastrointestinal tract we see a significant decrease in the ability of *Salmonella* to colonize. Interestingly, Willing *et al*. have recently reported a decrease in the prevalence of *Ruminococcaceae* in patients with Crohn’s disease [23] a condition associated with increased intestinal neutrophil recruitment [24]. Supporting our findings, these data suggest that neutrophil activity inhibits *Ruminococcaceae* growth in the gut.

Evidence from the last few years has brought to light that the immune system and the microbiota are involved in a great deal of crosstalk. It is no longer possible to ignore the microbiota when carrying out immunological research. A key example is the finding that mice from two different providers have different microbiota profiles and as a result have a different composition of Th17 and Treg cells [25]. We attempted to re-capitulate our model in neutrophil elastase knockout mice housed in a barrier facility at The Jackson Laboratories. Although we used wild-type control mice, also from this provider, and allowed the mice to acclimatize to our facility for five weeks, we found that the microbiota of naïve knockout mice was drastically different from that of naive wild-type mice (data not shown). It is impossible to understand what other effects could influence the microbiota composition in the creation of the knockout mice. Hence our loss of function and gain of function experiments that utilize the same strain of mice (wild-type) provides us with a better understanding of the impact of neutrophil elastase on the microbiota.

Infections in the gastrointestinal tract involve a complex relationship between the invading pathogen, the resident microbiota and the host immune response. Recent studies by our group and others have highlighted that these interactions are not binary, but interdependent in nature [8], [9], [12], [21], [26], [27], [28]. Here, we describe the identification of a new host factor that controls interactions between the host and the intestinal microbiota, which consequently alters pathogen interactions with the gut microbiota as well as the host. The microbiota is an integral part of the host immune response and it is becoming clear that we cannot discount it when attempting to understand the response generated towards pathogens. This study shows for the first time that neutrophil elastase is an important factor utilized by *Salmonella* to alter the microbiota profile and create a more favorable environment for its own colonization. These results extend our understanding of the role of the microbiota in mucosal infections. Further studies in this area will provide us with a magnified view of the intricate interplay between host, commensals and pathogens.

## Materials and Methods

### Ethics Statement

All animal work was approved and conducted in direct accordance with guidelines drafted by the University of British Columbia's Animal Care Committee and the Canadian Council on the Use of Laboratory Animals.

### Mice

Six- to eight-week-old female C57BL/6 mice (Jackson Laboratory, Bar Harbor, USA) were housed in the animal facility at the University of British Columbia in direct accordance with guidelines drafted by the University of British Columbia's Animal Care Committee and the Canadian Council on the Use of Laboratory Animals. Mice were fed a standard sterile chow diet (Laboratory Rodent Diet 5001; Purina Mills, St. Louis, USA) ad libitum throughout the experiments. Mice followed a 12 hour day and 12 hour night schedule, and were maintained under standard temperature controlled conditions.

### Bacterial Strains

*Salmonella enterica* serovar Typhimurium strains were all derivatives of SL1344 and were grown in Luria-Bertani medium at 37°C with shaking. The strains used had mutations on *aroA*, *aroAinvA* or *aroAssaR*.

### Antibiotic Treatment and Mouse Infections

Mice were treated with 450 mg/L of streptomycin (Sigma-Aldrich, St. Louis, USA) in drinking water as described previously [12]. Control mice were given sterilized, non-acidified drinking water without the antibiotic. After two days the antibiotic was withdrawn and mice were infected with the appropriate strain of *Salmonella* at 2.7×10^8^ cells/mouse by oral gavage. Uninfected control mice were given 100 µL of sterile LB broth. At five days post-infection mice were euthanized by CO_2_ asphyxiation and tissues were harvested aseptically for further evaluation.

### Tissue Collection and *Salmonella* Enumeration

Ceca were collected in 1 mL of sterile PBS on ice and homogenized with a MixerMill 301 (Retsch, Newtown, USA). Serial dilutions of the homogenates were plated on LB agar plates containing 100 µg/mL streptomycin to enumerate *Salmonella* colonization. The limit of detection of this assay is 100 colony-forming units per gram of cecum.

### Histopathology

Caecal samples were collected and fixed in 10% neutral buffered formalin overnight and then placed into 75% ethanol. Fixed tissues were embedded in paraffin, cut into 5-µm sections and stained with hematoxylin and eosin using standard techniques at the University of British Columbia Histology Laboratory. Pathological scores were assigned as previously described [22].

### SYBR Green Staining

SYBR Green (Invitrogen, Carlsbad, USA) stain, which binds to dsDNA, was used to determine the total numbers of intestinal microbes in the samples, as described previously [12]. A 1∶10 dilution of the homogenized cecal samples was fixed and stored in 3.7% formalin at 4 degrees until SYBR straining was carried out. Briefly, 2 µL of the fixed samples were stained with 0.25 µL SYBR Green (Invitrogen) and viewed with an Olympus 1×81 microscope. Three fields were randomly chosen and the numbers of cells were counted and averaged. The counts were made in a microscope field of a known diameter and corrected to the volume of sample used.

### Terminal Restriction Fragment Polymorphism (TRFLP) Analysis

TRFLP was used to evaluate microbiota community profiles as described previously [11]. Genomic DNA was isolated from murine feces using the QIAamp DNA stool minikit (Qiagen, Hilden, Germany) according to manufacturer’s instructions with the addition of a bead-beating step. Bacterial 16S rRNA genes were amplified with primers 8F (FAM labeled) (5′-AGAGTTTGATCMTGGCTCAG-3′) and 926R (5′-CCGTCAATTCCTTTRAGTTT’-3′). Amplicons were digested with MspI (New England Biolabs, Ipswich, USA) for 3 hours at 37°C. Samples were diluted 1∶10 and sent to the Nucleic Acid Protein Service Unit (University of British Columbia) for analysis on a 3730 DNA Analyser with a GeneScan 1200 LIZ size standard (Applied Biosystems, Foster City, USA). The profiles were imported into the GeneMarker v1.75 software (SoftGenetics, State College, USA) and fragment lengths were determined using the internal size standard and local Southern algorithm. Similarities of the microbial community structures between groups were determined by NMS analysis of the normalized data set using PC-ORD 5.0 and Past softwares. Similarities between treatment groups were calculated using Manhattan metrics, where individual sample TRF profiles were compared to the average TRF profile of the group indicated [29], [30].

### 16S rRNA Gene Pyrosequencing

For the identification of bacteria affected by infection and neutrophil elastase treatment, bacterial 16S rRNA gene fragments were amplified using 33 nucleotide-bar-coded primer pairs (27F; 5′-AGAGTTTGATCMTGGCTCAG-3′), (519R; 5′-GWATTACCGCGGCKGCTG-3′). PCR products were gel-purified (illustra GFX PCR DNA and Gel Band Purification Kit, Buckinghamshire, UK), pooled and pyrosequenced using a 454 Titanium platform (Roche, Branford, CT). Pyrosequencing reads were denoised using PYRONOISE [31], followed by extraction of variable regions V1 and V2 with V-XTRACTOR [32]. Chimeras were removed using UCHIME [33] and remaining sequences clustered into Operational Taxonomic Units (OTUs) using CROP [34]. Representative sequences from each OTU were classified using MOTHUR [35] by running them against the new GREENGENES database [36] and were taxonomically assigned with a minimum bootstrap support of 60%.

### Neutrophil Depletion

To deplete neutrophils, mice were treated with Ly6G/Ly6C antibody as previously described [37]. Briefly, mice were treated with 100 µg intraperitoneally on days -1, 1 and 3 post infection. Control mice were treated with PBS only. Neutrophil depletion was confirmed using FACS.

### Neutrophil Elastase Inhibition

In order to neutralize neutrophil elastase we used Sivelestat sodium salt hydrate (Santa Cruz Biotechnology Inc., Santa Cruz, USA) as previously described [38]. Briefly, mice were treated with 1 mg of the compound intraperitoneally two times each day on days 0, 1, 2, 3 and 4 post infection. Control mice were treated with PBS only.

### Recombinant Neutrophil Elastase Treatment

Mice were treated with recombinant neutrophil elastase (Athens Research and Technology, Athens, USA) on days 0, 1, 2, 3 and 4 post infection. Mice were given a total of 25 µg each day divided via oral and intraperitoneal injections. In order to ensure recombinant neutrophil elastase was present in the cecum during infection, ceca were collected at day 5 post infection and homogenates were assessed for neutrophil elastase presence using Western Blots.

### Flow Cytometry

Ceca were harvested from mice 5 days post infection and single cell suspensions were prepared. Briefly, ceca were finely chopped in RPMI and incubated with collagenase IV (Sigma-Aldrich) shaking for 2 hours at 37°C. Single cell suspensions were then isolated and staining was performed using CD45, CD3, NK1.1, GRI, MPO, CD11b, CD11c, MHCII, B220 fluorochrome-conjugated antibodies. Samples were run on an LSRII machine (BD Biosciences, Franklin Lakes, USA) and data was analyzed using FlowJo software.

### Statistical Analysis

Statistical significance was calculated using the Mann-Whitney test with a 95% confidence interval. All analyses were performed using GraphPad Prism version 4.0 (GraphPad Software, San Diego, USA). The results were expressed as mean values with standard errors of the means. Results were considered significantly different at *p*<0.05. Outliers were detected using the Grubbs’ test and removed from data sets when indicated.
